# Pani Profesor Zw. Dr Hab. n. Med. Krystyna Bożkowa - Redaktor Naczelny Developmental Period Medicine Medycyna Wieku Rozwojowego Zmarła w Dn. 29. Lipca 2018 Roku

**DOI:** 10.34763/devperiodmed.20182203.217218

**Published:** 2018-10-04

**Authors:** Ewa Helwich

**Affiliations:** 1 Kierownik Kliniki Neonatologii i Intensywnej Terapii Instytut Matki i Dziecka Redaktor tematyczny, Warsaw, Poland


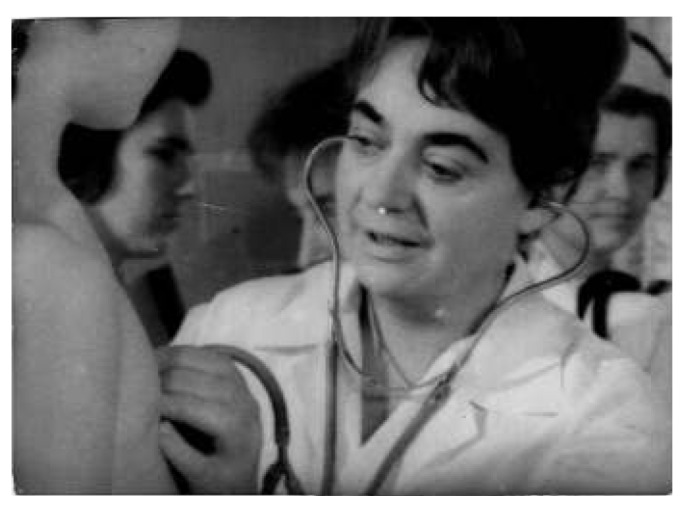


Pani Profesor Krystyna Bożkowa była na tyle bogatą postacią, że nie sposób jest zakreślić ramy tej charakterystyki. Powiedzieć, że jest lekarzem, nauczycielem, naukowcem i społecznikiem to zdecydowanie za mało.

W Instytucie Matki i Dziecka pracowała od 1960 r, kolejno na stanowiskach kierownika Kliniki Pediatrii, z-cy Dyrektora IMiD ds. naukowo-badawczych (9 lat) i wreszcie Dyrektora Instytutu (20 lat). Jest twórcą oryginalnej polskiej koncepcji medycyny wieku rozwojowego, integrującej specjalności pediatryczne. Jej zasługą jest konsekwentne wcielanie tej koncepcji w życie. Konsekwencja – to cecha, którą z pewnością Pani Profesor posiadała i której uczyła swoich asystentów, czego i ja doświadczyłam. „Jeśli powiedziałeś A, musisz powiedzieć Bi Ci tak dalej. Jeśli obroniłeś doktorat, następnego dnia zacznij planować habilitację, nie zatrzymuj się, szkoda czasu, idź dalej”.

W bogatej działalności naukowej Pani Profesor Bożkowej można wyodrębnić 4 główne kierunki badań: rozwój funkcji biochemicznych organizmu dziecka, reaktywność ustroju rosnącego, zwłaszcza na leki, wrodzone choroby metaboliczne i żywienie. Dziś możemy powiedzieć, żeta wizja sprawdziła się w pełni. Od lat 70-80. zeszłego stulecia stale poszerzany jest w IMiD panel badań przesiewowych wykonywanych u wszystkich noworodków w Polsce, opracowywany pod względem merytorycznym, metodycznym i organizacyjnym. W kolejnych pokoleniach dziesiątki dzieci zdiagnozowanych w okresie bezobjawowym i uchronionych przed ciężkimi uszkodzeniami których, jeśli już zaistnieją, nie da się odwrócić. To wszystko zaczęło się w czasach przaśnych, nieporównywalnych do tego, co jest naszym dzisiejszym doświadczeniem. Nie istniały granty badawcze ani stypendia naukowe, ale Pani Profesor potra+ła uzyskać dla Instytutu współpracę amerykańską, która umożliwiła zaczątki pediatrii metabolicznej w Polsce.

Pani Profesor Bożkowa potra+ła wydobyć z doniesień światowych to, co budowało nowe trendy w ramach medycyny wieku rozwojowego i zainteresować nimi swoich współpracowników. Szczególnie interesowała się tym, co w różnych specjalnościach medycznych dotyczyło mukowiscydozy. Cieszyła się ogromnie tym, że wreszcie w Polsce, w Instytucie Matki i Dziecka powstał ośrodek leczenia tej choroby na miarę XXI wieku.

Wielki dorobek dydaktyczny Pani Profesor wyraża się szkoleniem specjalistycznym ordynatorów oddziałów pediatrycznych w kraju oraz lekarzy z zagranicy na międzynarodowych kursach organizowanych w IMiD we współpracy ze Światową Organizacją Zdrowia. Oczami młodego wówczas lekarza patrzyłam na goszczących w Instytucie pracowników ochrony zdrowia z różnych stron świata, także z Afryki, w tradycyjnie kolorowych sukniach i zawojach na głowach. Na tle naszej szarej wówczas rzeczywistości wyglądali jak rajskie ptaki. Profesor Bożkowa stworzyła szkołę pediatryczną, specjalizując wielu lekarzy, kierując licznymi przewodami doktorskimi i habilitacyjnymi. Cechą tej szkoły był wysoki poziom fachowy (zawsze była bardzo wymagającym szefem i nie uznawała taryfy ulgowej bez względu na okoliczności), umiejętność pracy zespołowej („to dobrze, że są różne zdania, łatwiej jest uniknąć skrajności, ale musimy dojść do konsensusu”) oraz humanistyczny typ lekarza spo-łecznika („cóż znaczą finanse wobec misji, jaką mamy do wypełnienia”). Nieustannie dopingowała nas lekarzy w trakcie specjalizacji i młodych doktorantów do wytężonej pracy: „przecież poprzestanie na badaniu pacjentów i wydawaniu zaleceń to zbyt mało, to wkrótce każdego znudzi. Trzeba analizować to, z czego buduje się własne doświadczenie i pisać, pisać, pisać. Dopóki nie napiszesz, myślisz, że wszystko wiesz, a dopiero jak zaczniesz pisać, okaże się, ile tu jeszcze znaków zapytania pozostało do rozstrzygnięcia. A jak czasem uda ci się wymyśleć lub odnaleźć coś naprawdę oryginalnego, poczujesz radość twórcy i prawdziwego badacza”. I jeszcze jedno wspomnienie: kiedy ktoś narzekał, że z tym lub z tamtym profesorem trudno się porozumieć, Pani Profesor mawiała: „wszyscy profesorowie są trudnymi ludźmi, każdy na swój sposób, bo żeby dobrnąć do nominacji profesorskiej wręczanej przez Prezydenta RP trzeba pokonać tyle trudności, wykazać taki hart ducha, że to nie może pozostać bez śladu”.

Pani Profesor Bożkowa była koordynatorem Centralnego Programu Badawczo-Rozwojowego „Ochrona zdrowia matki, dziecka i rodziny” i koordynatorem międzynarodowym w zakresie pediatrii krajów socjalistycznych. Jako specjalista krajowy w zakresie ochrony zdrowia dzieci i młodzieży, a także jako dyrektor Instytutu była Profesor Bożkowa niestrudzonym adwokatem dzieci w zabezpieczaniu ich potrzeb zdrowotnych i społecznych. Pełniła wiele funkcji w licznych towarzystwach naukowych. Była ekspertem Światowej Organizacji Zdrowia i członkiem honorowym wielu towarzystw naukowych krajowych i zagranicznych, członkiem Komitetów i Komisji PAN, wielu Rad Naukowych. Była aktywnym działaczem społecznym – m.in. w Rządowej Komisji Ludnościowej, w Krajowej Radzie Kobiet.

Od 1996 roku Pani Profesor Bożkowa była Redaktorem Naczelnym *Developmental Period Medicine/Medycyny Wieku Rozwojowego*, czasopisma, które jest kontynuacją poprzednich czasopism Instytutu Matki i Dziecka.

Dzięki doskonałej znajomości warsztatu naukowego Pani Profesor inspirowała autorów do zajęcia się najnowszymi tematami z zakresu medycyny wieku rozwojowego. Szczególnie ceniła badania interdyscyplinarne oraz podkreślała znaczenie publikowania ich rezultatów.

Pochylała się nad każdą pracą, wkładając wiele wysiłku by była doskonała zarówno merytorycznie jak i językowo. Wszystko to przyczyniło się do tego, że czasopismo jest jednym z bardziej liczących się w Polsce.

Półtora roku temu, z okazji 65. rocznicy istnienia Instytutu Matki i Dziecka jednym z punktów programu była laudacja, podziękowanie Pani Profesor Bożkowej za lata Jej owocnej pracy na rzecz polskiej pediatrii. Dziś myślę, jak dobrze się stało, że zdołaliśmy to zrobić wtedy, gdy Pani Profesor była jeszcze z nami. W ostatnich latach spotykaliśmy Panią Profesor na posiedzeniach Rady Naukowej i w pokoju redakcyjnym czasopisma *Developmental Period Medicine/Medycyna Wieku Rozwojowego*. Ciągle zadziwiała nas bystrością swego umysłu i gotowością dyskusji o nauce, jej sukcesach i zagrożeniach. Potrafiła zarażać nas swoim entuzjazmem i chęcią odkrywania tego, co jeszcze przed nami. Tym wszystkim sprawiła, że dziś czujemy się osieroceni.

